# Serological Assessment of Alpha Galactose, N-Glycolylneuraminic Acid, and Histopathological Observations in Xenograft Recipients

**DOI:** 10.7759/cureus.91236

**Published:** 2025-08-29

**Authors:** Balasundari Ramesh, Sowmya Ramanan, Balaji Srimurugan, Adegbenro O Fakoya

**Affiliations:** 1 Cellular Biology and Anatomy, Louisiana State University Health Sciences Center, Shreveport, USA; 2 Cardiothoracic Surgery, Sree Chitra Tirunal Institute for Medical Sciences and Technology, Trivandrum, IND; 3 Cardiovascular Surgery, Amrita Institute of Medical Sciences and Research Centre, Chennai, IND

**Keywords:** alpha galactosidase (α-gal), biomaterial, cd19, decellularization, n-glycolylneuraminic acid (neu5gc), smooth muscle actin, von williebrand factor, xenograft

## Abstract

Background and aims

The galactose-α-1,3-galactose (αGal) epitope and the associated anti-Gal antibodies, along with the non-human sialic acid N-glycolylneuraminic acid (Neu5Gc) and its corresponding anti-Neu5Gc antibodies, represent critical obstacles in the field of xenotransplantation. We present an evaluation of serological and histopathological data from patients who experienced immunological rejection after receiving decellularized xenografts. This study aims to analyze the long-term immune responses that occur following the implantation of these grafts, providing insights into the mechanisms driving rejection and their potential impact on transplant outcomes.

Methods

Decellularized xenografts, such as bovine pericardium and porcine pulmonary artery treated with a novel patented processing technique, were utilized in cardiovascular surgeries over the course of a decade. Serum samples from patients following xenotransplantation were examined for α-Gal and Neu5Gc titers after various follow-up periods. When explanted during later surgeries, the xenograft materials were assessed histologically using hematoxylin and eosin (H&E), von Kossa, and Movat's pentachrome staining. Immunohistochemical staining for von Willebrand Factor (vWF), alpha-smooth muscle actin (αSMA), and B lymphocyte antigen CD19 was performed to evaluate endothelialization, calcification, fibrosis, and immune rejection. No healthy volunteers were enrolled as study participants. However, for the purpose of baseline serological comparison, serum samples from one healthy adult blood donor (33/M) were used as the standard. This donor had no history of xenograft implantation or known immune disorders.

Results and conclusion

Histological evaluation showed human cell infiltration within the decellularized xenograft scaffold, with evidence of reendothelialization on the luminal surface. The neointimal layer had variable thickness, with aligned collagen fibers. Early remodeling was indicated by minimal fibroblastic invasion and the presence of microcapillaries. von Kossa staining revealed insignificant calcium deposits, and immunohistochemistry showed minimal signs of immune rejection. Serological analysis of seven patients revealed varying responses to α-Gal and Neu5Gc. The patients had a mean age of 16.57 years (ranging from three to 52 years) and a mean follow-up period of 6.14 years (ranging from four to nine years) at the time of blood sampling. The study included four patients with bovine pericardium grafts, one with a bovine jugular vein graft, and two with porcine pulmonary artery grafts. Anti-αGal antibody levels were consistent across all patients, while two patients showed no anti-Neu5Gc antibodies. The remaining five patients exhibited titers with a high standard deviation.

This in vitro evaluation indicates that decellularized xenografts processed using the patented technology provoke minimal tissue and immune responses, making them safe and durable options for cardiovascular remodeling surgeries.

## Introduction

Xenogenic antigens remain a significant barrier to the clinical use of xenografts. Approximately 28 million years ago, an evolutionary event in catarrhines (humans, apes, and Old-World monkeys) led to the inactivation of the α1,3-galactosyltransferase (GGTA1) gene, resulting in the loss of the α-Gal epitope [1-3]. Another key antigen, N-glycolylneuraminic acid (Neu5Gc), is expressed in most animals, including pigs and Old-World non-human primates, but is absent in humans, New World monkeys, ferrets, and platypuses, with a low incidence in birds and reptiles [4,5]. Human tissues do not produce Neu5Gc due to the inactivation of the sialic acid-modifying enzyme cytidine monophospho-N-acetylneuraminic acid hydroxylase (CMAH) gene during hominid evolution [6,7]. As a result, humans develop anti-α-Gal and anti-Neu5Gc antibodies, particularly IgG, when exposed to xenogenic cells [8].

The chronic shortage of cardiovascular homografts, along with issues such as donor size mismatches, morbidity, and high failure rates of cryopreserved grafts, has driven the development of xenograft-based tissue-engineered grafts and genetically modified porcine hearts for transplantation [9,10]. Most artificial scaffolds fail to provide the required tensile strength and bioactive extracellular matrix (ECM) support needed for effective cell-homing properties [11,12]. The ideal scaffold for reconstructive surgeries would be a decellularized ECM from the target tissue, as the decellularization process removes donor cell antigenicity while maintaining an adequate 3D structure and molecular composition [13,14]. Over the past decades, decellularized xenograft scaffolds derived from porcine and bovine tissues have been widely used in cardiovascular applications, such as repairing septal and valvular defects.

This study investigates the presence of the xenoantigens α-Gal and Neu5Gc in patients with decellularized xenograft implants using a serum-based approach, including immunohistochemistry on the explanted xenografts. This in vitro detection method allows for the direct assessment of xenograft immunogenic rejection by human serum, enabling monitoring of xenograft recipients for both acute and chronic rejection.

## Materials and methods

Harvesting and processing of xenografts

Bovine and porcine xenografts were harvested under sterile precautions from an inspected abattoir. Approval from the Institutional Animal Ethics Committee was not required for the procurement of xenogeneic tissues, as the bovine and porcine materials were obtained from animals slaughtered for commercial meat production, following the guidelines set forth by the Committee for the Purpose of Control and Supervision of Experiments on Animals (CPCSEA), Government of India. However, all procedures related to the development, testing, and evaluation of the grafts were reviewed and approved by the appropriate Institutional Ethics Committee to ensure compliance with ethical and biosafety standards. The harvested samples were collected with sterile precautions in Hanks Balanced Salt Solution (HBSS, Himedia TS1003) solution with a cocktail of antibiotics (Himedia, A002) and transported to the lab with icepacks. The samples were subjected to pre-sterility tests for bacteria, fungi, and viruses. The microbe-free tissue samples were dissected in a laminar air hood to remove unwanted fat and adhering tissues. The dissected xenografts were subjected to decellularization with sodium deoxycholate and nucleases. Subsequently, they were subjected to crosslinking with formaldehyde. Furthermore, processing such as anti-thrombogenic by immersing the grafts in heparin solution (50 IU/mL phosphate buffered saline (PBS)) to inhibit platelet adhesion and thrombus formation and anti-calcification treatments was performed using the aldehyde capping method, wherein grafts were treated with 1% w/v L-glutamic acid to covalently bind and neutralize residual free aldehyde groups remaining after crosslinking process, thereby reducing the risk of calcific degeneration. Finally, the processed xenografts were preserved in 70% alcohol and stored at 4-8°C. The processing protocol and the composition of the preservative used for storing the tissue are patented (patent no: US2012/0029655A1).

The processed xenografts were subjected to various validations: (1) Hematoxylin and eosin (H&E) stain to check the acellularity and collagen architecture; (2) DNA extraction and agarose gel electrophoresis to confirm the removal of cells by analyzing the presence of DNA after decellularization; (3) collagenase digestion test and hydroxyproline assay for efficacy of crosslinking; (4) mechanical testing for evaluating mechanical properties of tissues as per ISO 5840-1/3: Tensile strength, in vitro real-time fatigue testing, suture retention, and burst strength; (5) scanning electron microscopy (SEM) and confocal microscopy for evaluating the architecture (extracellular matrix structure); (6) Fourier transform infrared spectroscopy (FTIR) to assess resident collagen; (7) differential scanning calorimetry (DSC) for validation of the heat stability of the grafts; (8) microbial sterility for bacteria, fungi, and viruses; (9) thrombogenicity test; (10) biocompatibility as per ISO 10993-1:2018: In vitro cytotoxicity test using BALB/3T3 cell line, systemic toxicity studies in albino mice, and hypersensitization studies in guinea pigs; (11) large animal implantation in Madras Red breed sheep; (12) histological analysis of explanted tissues. These validations were done to confirm the safety of xenografts for human applications.

Clinical trials

This clinical trial was performed by Frontier Lifeline Pvt Ltd (2016-2018), with regulatory clearance from the Drug Controller, Government of India (DCGI) obtained for human clinical trials as an interventional single-arm multicenter trial. All the trials were conducted following the Declaration of Helsinki after the ethics committee approval (CTRI/2017/08/009225). The processed xenografts were prewashed twice with heparin 14 IU/ml of normal saline before surgical implantation. A volume of 10 ml of blood was drawn postoperatively from the patients for serological analysis during the follow-up (Table 1).

**Table 1:** Serum and explanted xenograft details Clinical and surgical details of patients from whom serum samples (Investigational blood IB01-07), one explanted xenograft tissue were obtained and one standard serum from healthy donor. All study samples were derived from patients who had undergone prior cardiac surgeries involving bovine or porcine valve/conduit implants. Ages are given in years; M=male, F=female. TOF: Tetralogy of Fallot; PA: pulmonary atresia; BAV: bicuspid aortic valve; AS: aortic stenosis; PR: pulmonary regurgitation; DORV: double outlet right ventricle; LPA: left pulmonary artery; ICR: intracardiac repair; RV: right ventricle; MAPCAs: major aortopulmonary collateral arteries; TR: tricuspid regurgitation; VSD: ventricular septal defect.

S.No	Sample Code	Age and Sex	Blood group	Surgery
1	IB01	10/M	B+	TOF with Pulmonary Atresia, RV -PA conduit with hand-sewn bovine pericardium conduit (2012 May). On follow-up - asymptomatic
2	IB02	52/M	B+	BAV with severe AS, Ross procedure; Pulmonary autograft was implanted into the aortic position and porcine pulmonary artery xenograft was implanted in 2006, on follow-up – asymptomatic, Mild PR.
3	IB03	9/M	O+	DORV, pulmonary atresia with LPA stenosis BD Glenn with PA plasty with bovine pericardium (2005).Underwent completion Fontan in 2014
4	IB04	13/M	O+	TOF, absent pulmonary valve, absent LPA, S/P ICR & RV PA conduit with bovine IJV (2005) (presented with severe PR with conduit dilatation). Underwent redo surgery for Conduit replacement with homograft (2014)
5	IB05	3/F	B+	Truncus arteriosus repair done in 2011 ( hand-sewn bovine pericardial 11 mm conduit used). Has severe conduit obstruction, now planned for PA stenting
6	IB06	15/F	B+	TOF with pulmonary atresia with severe LPA stenosis. Underwent ICR with bovine pericardium augmentation of the LPA (2007). Currently underwent redo surgery for residual VSD and severe TR. Current status: Convalescing well
7	IB07	14/M	O+	TOF with Pulmonary atresia with MAPCAs, Underwent ICR with RV-PA conduit with porcine pulmonary artery xenograft (2008). On follow-up - Asymptomatic
8	Explanted Xenograft	4.5/M	B+	Unifocalization and RV to PA conduit with indigenous decellularized bovine pericardium. Explanted in 2014
9	Standard for Serology	33/M	B+	Healthy blood donor

Inclusion and exclusion criteria

The study included patients who had undergone cardiovascular surgery using decellularized xenograft materials such as bovine pericardium, bovine jugular vein, or porcine pulmonary artery, all processed using a patented decellularization technology. Eligible participants were those with a minimum postoperative follow-up duration of four years and for whom serum samples were available for serological analysis: (1) tetralogy of Fallot (TOF) with pulmonary atresia - ventricular septal defect (VSD); (2) double outlet right ventricle (DORV) with pulmonary atresia; (3) reoperations for patients operated for TOF requiring pulmonary valve replacement; (4) truncus arteriosus; and (5) TOF with absent pulmonary valve; (6) Ross procedure (the right ventricular outflow tract (RVOT) reconstruction). Additionally, patients who had undergone reoperation and whose explanted grafts were available for histological and immunohistochemical evaluation were included. Informed consent for the use of clinical data and biological specimens in research was a prerequisite for participation.

Patients were excluded if they had incomplete clinical documentation, a follow-up period of less than four years, or lacked accessible serum or tissue samples. Individuals who had received xenografts processed using alternative decellularization techniques were also excluded to maintain consistency in graft preparation. Furthermore, patients with known autoimmune diseases, immunodeficiencies, or those undergoing immunosuppressive therapy were excluded due to the potential for confounding immune response data. Any patient who declined or withdrew consent was not considered for inclusion.

Enzyme-linked immunosorbent assays (ELISA)

The ELISA technique was used to measure anti-α-Gal IgG and anti-Neu5Gc in patients at six months after cardiac surgery. Galα1.3-Galβ1-4GlcNAc-R (44711, Sigma Aldrich, St Louis, MI, USA), a solid-phase synthetic antigen 10 µg/mL, and Neu5Gc (Sigma Aldrich, G9793), a solid-phase semi-synthetic antigen 10 µg/mL, were dissolved in 50 mM sodium carbonate-bicarbonate buffer, pH 9.5. These antigens were coated in triplicate in 96-well microtiter plates (Costar, Arlington County, VA, USA) at 1 µg/mL of methanol. The methanol was allowed to evaporate completely for 4 h at room temperature (RT), and plates were incubated overnight at 4°C. The blocking was done with 1% ovalbumin in phosphate-buffered saline (PBS) blocking buffer, pH 7.5, for two hours. After incubation with human serum, the samples were diluted 1:100 in the same blocking solution for 4 h at RT. The plates were washed three times with PBS containing 0.1% Tween (PBST) and subsequently incubated for one hour at RT with horseradish peroxidase (HRP)-conjugated detection antibodies, such as anti-human IgG-Fc (Sigma Aldrich, I9135) and anti-human IgG subclasses (Sigma Aldrich, A8667), diluted at a 1:200 ratio with PBS. After an hour and another washing step, a color reaction was obtained with peroxidase reagent tetramethyl benzidine (TMB, Sigma Aldrich ES001) and stopped with 2 N sulfuric acid. Optical density was read at 450 nm using a Victor3 plate reader (1420 Multilabel Counter, Perkin Elmer, Waltham, MA, USA). The serum from a healthy donor was used to compare measurements. Results were expressed as percentage increase/decrease, and preoperative values were set as 100%.

Valve histology and immunohistochemistry

The explanted decellularized pericardium (after four years) was fixed with 4% formaldehyde in 0.1 M phosphate buffer (pH 7.4), rinsed with solutions of sucrose in PBS (5%, 10%, 20% and 30%) and then frozen in liquid nitrogen. From this, 10 µm sections were cut on a cryostat microtome (Cryocut model 3000; Leica Biosystems, Nussloch, Germany) and mounted on gelatin-coated slides. These slides were stained with H&E, von Kossa, and pentachrome and subjected to immunohistochemical analysis. The sections were double fluorescence-labeled with antibodies against alpha SMA (1A4, Invitrogen part of Thermo Fisher Scientific, Waltham, MA, USA), CD19 (HIB19, Invitrogen) and von Willebrand factor ( vWF) (F8/86, Invitrogen) and 4′,6-diamidino-2-phenylindole (DAPI) against DNA of nucleated cells; sections of explanted valves were rinsed in PBS, secondary antibodies (1:500) was applied for two hours at 37°C. After rinsing, the sections were mounted with a mounting medium containing DAPI (Fluromount, Invitrogen). Labeled sections were analyzed, and photos were documented under a fluorescent microscope (Carl Zeiss, Oberkochen, Germany).

Statistical analysis

A statistical comparison of alpha-Gal and Neu5Gc antibody levels between xenograft recipients and a standard from a healthy volunteer was performed using SPSS software (SPSS for Windows Version 15, SPSS Inc., Chicago, IL, USA). Mann-Whitney U test and Wilcoxon test were used to calculate significance and a p-value. We had used only one healthy donor blood sample due to the difficulty in identifying an age-matched healthy donor for each sample who did not consume any red meat (pork and beef) and had no xenograft implantation and no history of immunological disorders.

## Results

**Figure 1:**
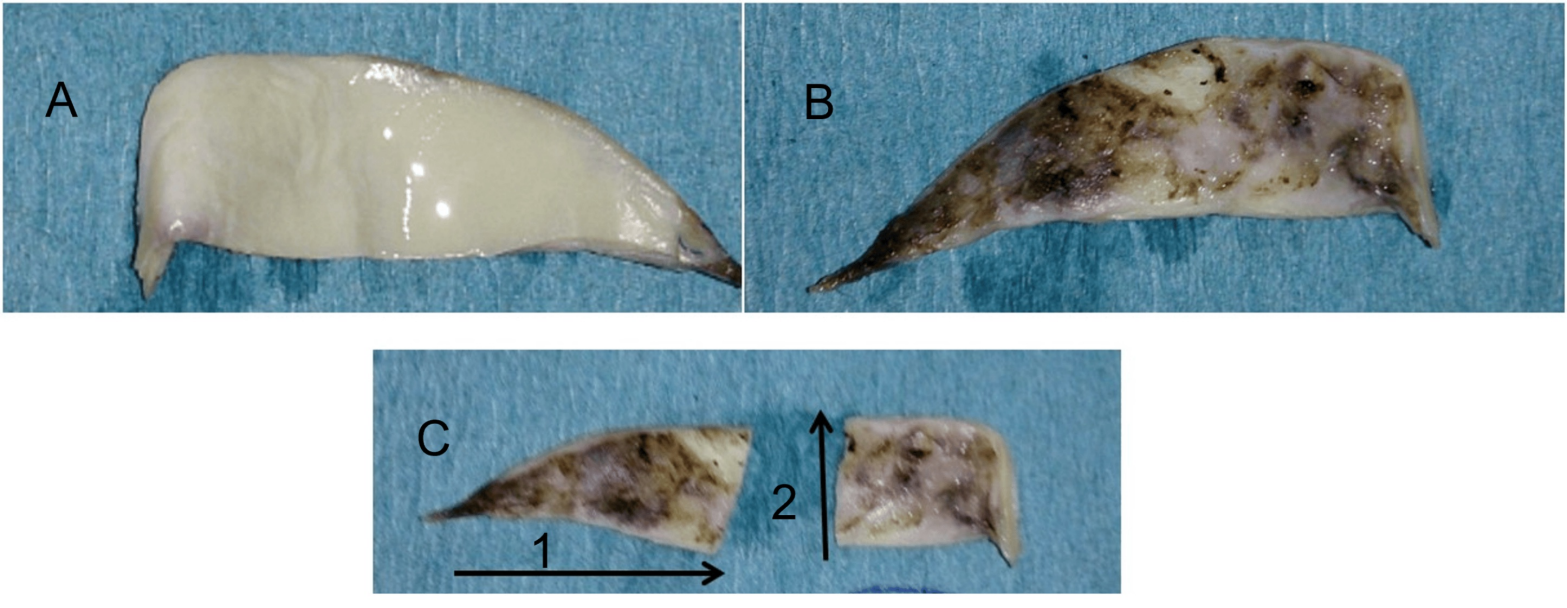
Gross Morphology of Explanted Bovine Pericardial Xenograft (A) Explanted bovine pericardium prior to showing smooth, glistening luminal surface. (B) Explanted bovine pericardial xenograft exterior surface demonstrating fibrotic thickening and surface discoloration suggestive of degeneration and calcification. (C) The explanted xenograft was sectioned for further analysis: region C1 represents the longitudinal orientation, while region C2 represents the cross-sectional orientation, both of which were subjected to histological and biochemical characterization.

**Figure 2:**
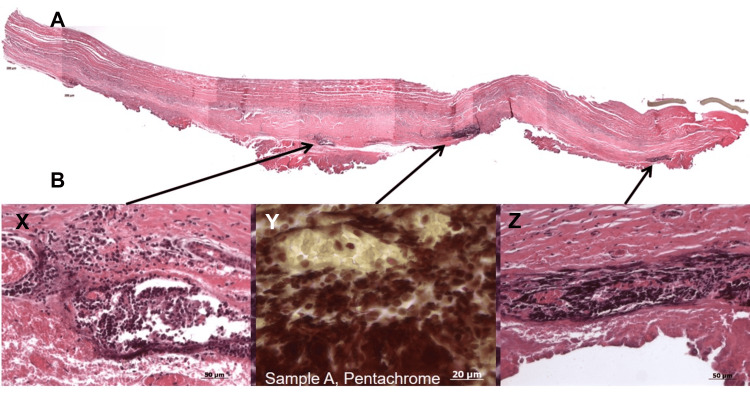
Histological Analysis of Explanted Bovine Pericardial Xenograft Low- and high-power views of explanted bovine pericardium stained with hematoxylin and eosin (H&E) and Pentachrome (middle panel). A: The low-power panoramic section (top panel) shows regions of structural degeneration and cellular infiltration. B: High-power images (X: bottom left and Z: right) demonstrate marked inflammatory infiltration and evidence of neovascularization (arrows). Y: Pentachrome staining (bottom center- labelled as Sample A is the C1 longitudinal section of the explanted tissue) highlights areas of early calcification and fibrosis. Scale bars: 50 µm (H&E); 20 µm (Pentachrome).

**Figure 3:**
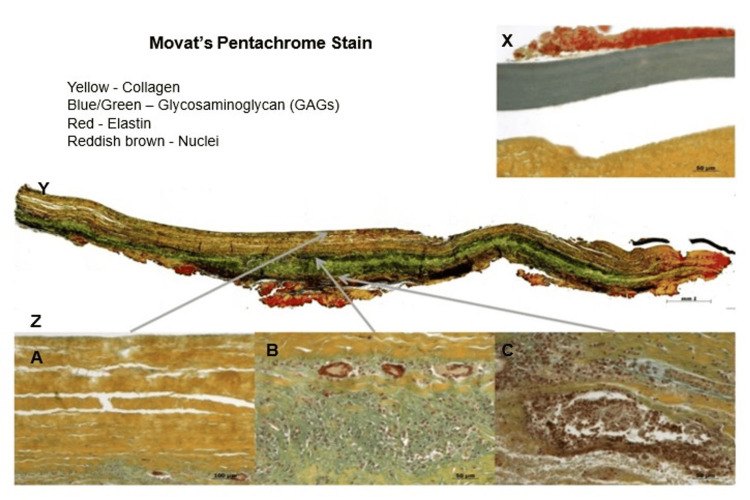
Pentachrome Staining of Explanted Bovine Pericardial Xenograft Pentachrome staining of the explanted bovine pericardium reveals preserved and organized extracellular matrix architecture. X: Yellow indicates collagen fibers; blue denotes glycosaminoglycans (GAGs); red highlights elastin fibers; and reddish-brown shows cellular nuclei. Y: Low magnification of pentachrome stained section. A, B, C: High-magnification insets illustrate regions of aligned collagen, abundant GAGs, elastin-rich zones, and newly formed capillaries indicative of neovascularization. Scale bars: 50 µm and 100 µm as indicated.

The processed xenografts were completely acellular, with no detectable traces of DNA, and retained scaffold properties similar to native tissue. Mechanical and biological testing demonstrated that they possessed both tensile strength and the desired physical and biocompatible characteristics. Additionally, they were sterile, non-cytotoxic, and fully biocompatible. The explanted bovine pericardial RV-PA conduit xenografts (Figure 1; explanted and fixed bovine pericardial tissue), analyzed with H&E and Movat's pentachrome staining, revealed collagen fibers in a longitudinal arrangement within the luminal layer, along with flat, endothelial-like cells (Figure 2). Pentachrome staining further showed the presence of endothelial cells, a neointimal layer with infiltrating cells, glycosaminoglycans (GAGs), and aligned collagen fibers. A high cell density was observed, extending from the tunica media to the adventitial side, with cell clusters adjacent to the adventitia. Microcapillaries containing erythrocytes were also seen in the adventitia (Figure 3).

**Figure 4:**
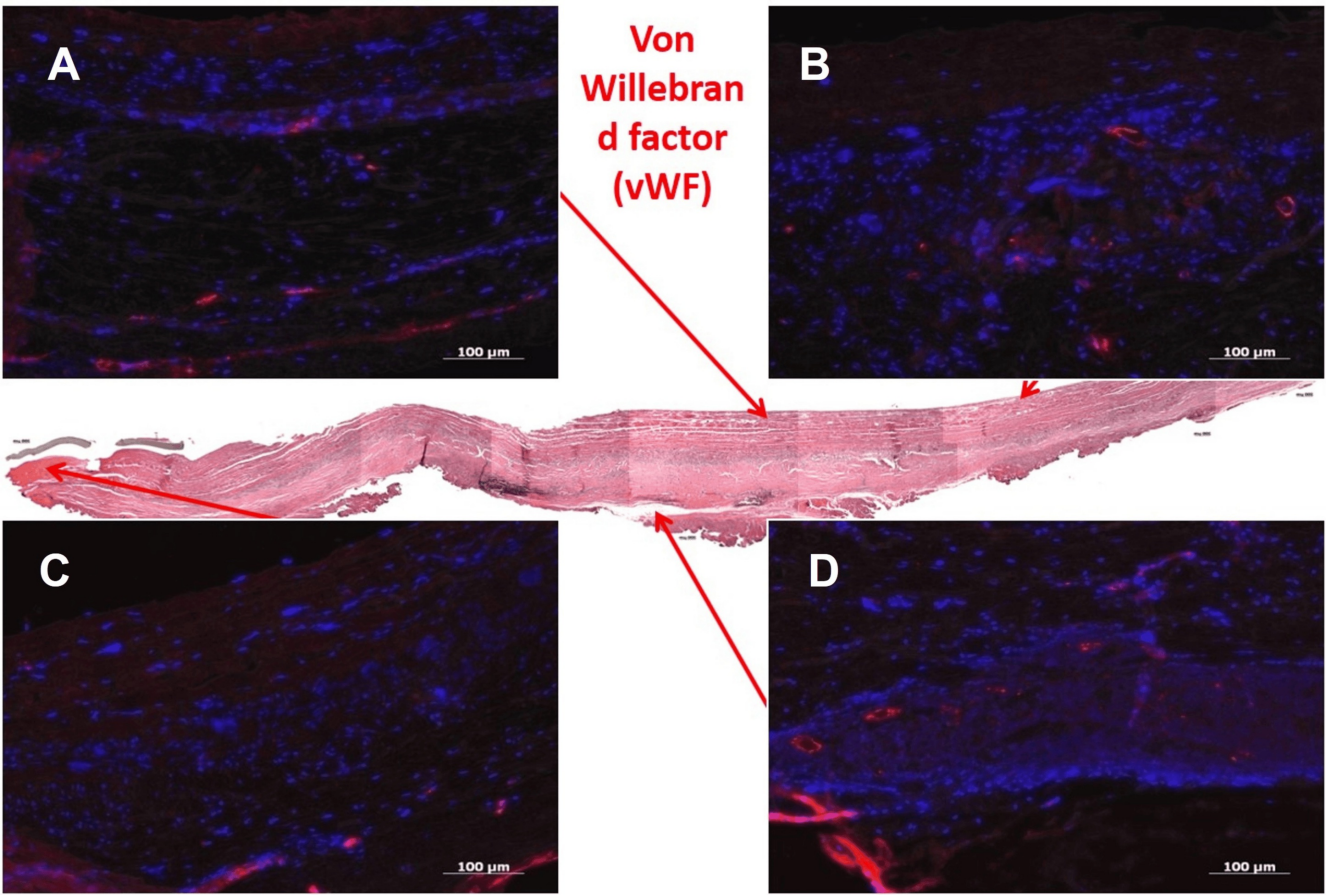
Immunohistochemical Staining of Explanted Bovine Pericardial Xenograft for von Willebrand Factor (vWF) A,B,C and D are the immunofluorescence staining demonstrating endothelial marker expression in the explanted bovine pericardium. Rhodamine-conjugated antibody labels vWF positive endothelial cells in red, indicating the presence of neovessels. Nuclear counterstaining with DAPI highlights cell nuclei in blue. The localization of vWF+ cells supports active neovascularization within the graft tissue. DAPI: 4′,6-Diamidino-2-phenylindole.

**Figure 5:**
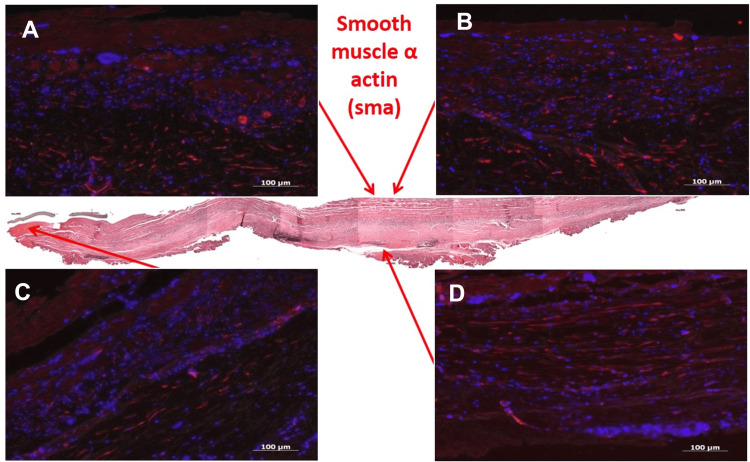
Immunohistochemical Staining of Explanted Bovine Pericardial Xenograft for Smooth Muscle Actin (SMA) A,B,C and D: Immunofluorescence staining shows expression of smooth muscle actin (SMA) in the explanted bovine pericardial tissue. SMA-positive cells appear red, indicating the presence of smooth muscle cells or myofibroblast-like cells within the graft. Nuclear staining with DAPI appears blue, confirming cellular localization. The presence of SMA+ cells suggests active tissue remodeling and vascular integration.

**Figure 6:**
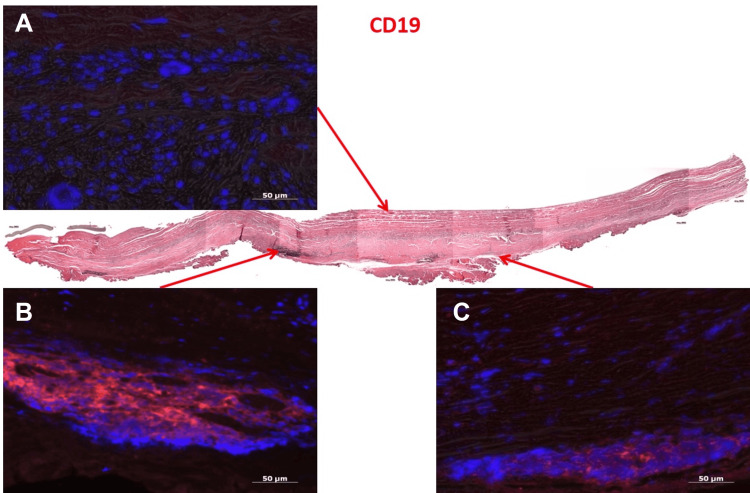
Immunohistochemical Staining of Explanted Bovine Pericardial Xenograft for CD19 A,B and C: Immunofluorescence staining reveals CD19 expression in the explanted bovine pericardial tissue. CD19-positive cells are visualized in red, indicating the presence of B lymphocytes within the graft. Nuclear staining with DAPI appears in blue, confirming cellular distribution. The presence of CD19+ cells suggests an ongoing immune response or lymphoid infiltration in the xenograft tissue.

Immunohistochemistry for vWF revealed vWF+ cell layers on the lumen and in microcapillaries within the adventitia (Figure 4). Smooth muscle actin staining was strongly positive in the tunica media, indicating the presence of myofibroblasts and smooth muscle cells (Figure 5). The adventitial side showed a mild positive reaction for CD19+ B cells, though the central longitudinal region was negative (Figure 6).

The mean age of the seven xenograft recipients in this serological study was 16.5 years, consisting of two women and five men (see Table 1: Sample code IB01-IB07). Two of the recipients had decellularized porcine pulmonary artery grafts, while five had decellularized bovine pericardium grafts. None of the serological study participants consumed pork or beef, including the standard serum from a healthy donor; their diets primarily consisted of lamb, chicken, and fish, without red meat. All had received vaccinations for Bacillus Calmette-Guérin (BCG), diphtheria, pertussis, tetanus (DPT), *Haemophilus influenzae* type B, hepatitis B, influenza, measles, mumps, rubella (MMR), oral poliovirus vaccine (OPV), typhoid, and varicella-zoster.

**Figure 7:**
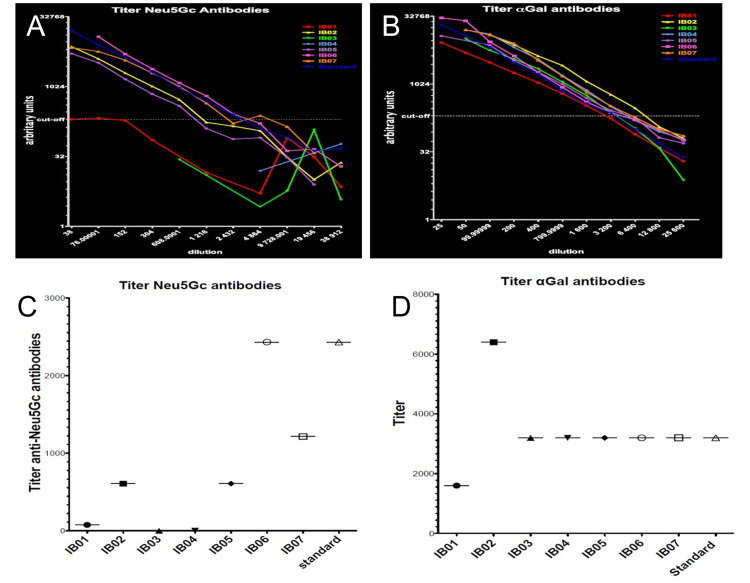
Serum Antibody Titers Against Neu5Gc and Alpha-Gal in Xenograft Recipients Compared to Standard The titer of Neu 5Gc and alpha Gal antibodies in comparison with standard (blue color). A: Neu5Gc antibodies arbitrary units at various dilution of serum, B: alpha gal antibodies arbitrary units at various dilution of serum. C: Titer of anti Neu5Gc antibodies from xenograft recipients compared with standard. D: Titer of anti-alpha Gal antibodies compared with standard.

The statistical analysis comparing antibody titers between xenograft recipients and a healthy control (Figure 7) revealed differing results based on the test applied. The Wilcoxon Signed-Rank Test, suitable for paired and non-normally distributed data, showed a statistically significant reduction in anti-Neu5Gc titers among patients compared to the healthy standard (p<0.05), suggesting a possible immune modulation following long-term xenograft implantation, as shown in Table 2. In contrast, no significant difference was observed in anti-αGal titers (p>0.05), indicating a relatively stable immune response to αGal antigens. On the other hand, the Mann-Whitney U test, a non-parametric test for independent groups, did not detect any statistically significant differences for either antibody type, as shown in Table 3. This discrepancy likely reflects the difference in test sensitivity and assumptions, particularly given the small sample size and paired design, which favors the Wilcoxon test for within-subject comparisons. Together, the findings highlight a selective reduction in anti-Neu5Gc antibodies in xenograft recipients and underscore the importance of choosing appropriate statistical methods based on data structure and sample pairing.

**Table 2:** Wilcoxon Signed-Rank Test Results The Wilcoxon test was used due to the non-normal distribution and small sample size. A p-value < 0.05 was considered statistically significant. αGal: Alpha galactosidase; Neu5Gc: N-glycolylneuraminic acid.

S.No	Antibody Type	Comparison (Patients vs. Healthy Standard)	Median Titer (Patients)	Standard Titer (Healthy Control)	p-value	Comments
1	Anti-Neu5Gc	Paired (n=7 vs. 1 Standard)	1800	2500	<0.05	Statistically significant difference
2	Anti-αGal	Paired (n=7 vs. 1 Standard)	4000	4000	>0.05	No significant difference

**Table 3:** Mann-Whitney U test Mann-Whitney U test comparing serum antibody levels against Neu5Gc and α-Gal between xenograft recipients (Sample code IB represents Investigational blood). αGal: Alpha galactosidase; Neu5Gc: N-glycolylneuraminic acid.

S.No	Antibody Type	Groups Compared	Sample Size	Patient Demographics (Age/Sex)	Median Antibody Titer	U Statistic	Comments
1	Anti-Neu5Gc	Patients vs. Healthy Standard	Patients (n=7), Standard (n=1)	IB01: 10/M	Patients: 1800	1.5	No statistically significant difference
				IB02: 52/M	Standard: 2500		
				IB03: 9/M			
				IB04: 13/M			
				IB05: 3/F			
				IB06: 15/F			
				IB07: 14/M			
2	Anti-αGal	Patients vs. Healthy Standard	Patients (n=7), Standard (n=1)	Same as above	Patients: 4000	3.5	No statistically significant difference
					Standard: 4000		

## Discussion

Xenogeneic tissues are currently employed in clinical practice to create biological substitutes (bioprosthetic heart valves) and in the repair of various damaged tissues (pericardium, gastric-mucosa, nerves, cartilage). Many studies have shown that xenogeneic tissues express superficial epitopes, such as alpha-Gal, capable of triggering hyperacute and acute vascular rejection phenomena. 

Porcine and bovine xenogeneic tissues offer versatile options for creating biological and bioprosthetic substitutes in cardiac surgery. However, a key challenge in the clinical application of xenografts is the expression of xenoantigens, which trigger immune rejection. In 1984, Galili et al. [2] characterized the xenoantigen "α-Gal" epitope (Galα1−3Galβ1−(3)4GlcNAc-R, where R is an underlying glycoconjugate). This epitope is widely expressed in most mammals except for Old World primates and humans [15]. Anti-α-Gal antibodies are introduced in humans soon after birth through exposure to gut bacteria bearing similar epitopes, complicating xenotransplantation [16]. The binding of human or primate anti-α-Gal antibodies to pig Gal epitopes leads to hyperacute rejection [17]. Similarly, Neu5Gc, a sialic acid molecule, is another xenoantigen absent in humans due to a mutation in the gene encoding CMP-N-acetylneuraminic acid hydroxylase [18].

Advances in gene editing technologies such as zinc finger nucleases, transcription activator-like effector nucleases, and clustered regularly interspaced short palindromic repeats (CRISPR) have enabled the development of pigs homozygous for α1,3-galactosyltransferase gene knockout (GTKO) [19] and cytidine monophosphate-N-acetylneuraminic acid hydroxylase gene knockout (Neu5GcKO) [20,21], facilitating successful pig-to-baboon organ transplantation. Prolonged survival times have recently been achieved in xenotransplantation of the heart (>900 days), kidney (>400 days), liver (29 days), islet (>600 days), and cornea (>389 days) using genetically engineered knockout pigs and newer co-stimulation blockade agents [19-25].

In 2022, Moazami et al. performed two orthotopic heart transplants using 10-gene-edited (10GE) porcine xenografts in brain-dead recipients. This trial allowed for the monitoring of graft function, xenoantibody injury, and frequent sampling of xenograft tissue, blood, and body fluids over three days, collecting valuable data without risk to the decedent recipient [26]. Further, in 2022 and 2023, two historic porcine-to-human heart transplants were conducted at the University of Maryland School of Medicine. Genetically modified pig hearts from Revivicor, which underwent 10GE, knocking down three immune rejection-related genes and adding six human genes along with one growth control gene, were successfully transplanted into 57- and 58-year-old men with end-stage heart disease. The patients survived for two months and six weeks, respectively. Gene editing to remove xenoantigens such as α1,3-Gal (encoded by GGTA1) and Neu5Gc (encoded by CMAH and B4GalNT2) is a critical strategy to reduce immune rejection [27].

In 2016, Hurh et al. [28] generated human embryonic kidney (HEK293) cells expressing xenogeneic Neu5Gc/α1,3-Gal antigens to investigate human antibody binding and complement-dependent cytotoxicity (CDC) using human sera. They discovered that both IgM and IgG bound to α1,3-Gal, while only IgG bound to Neu5Gc, with blood group A showing the highest degree of IgG binding to α1,3-Gal.

This serological study could be more comprehensive by analyzing antibody titers at intervals such as zero months, three months, six months, and one year post-xenograft implantation. While ABO blood group reactivity to these antigens and geographic comparisons were not feasible due to sample limitations, we successfully standardized serological protocols. Since bovine serum albumin, commonly used as a blocking agent in enzyme-linked immunosorbent assays (ELISAs), may contain Neu5Gc glycans [27,29], we opted to use chicken ovalbumin in our assay. This simplified protocol can be applied to investigate the presence of these antigens and antibodies in decellularized grafts or the serum of xenograft recipients.

We measured anti-Neu5Gc and anti-αGal antibody titers in seven patients (ages 3-52 years; five men, two women) following congenital heart surgeries involving bovine pericardium and porcine pulmonary artery xenografts. Anti‑Neu5Gc responses, which reflect immune reactivity to the foreign sialic acid Neu5Gc abundant in bovine and porcine bioprosthetic tissues [30] varied widely. The highest anti‑Neu5Gc titers were observed in younger patients, most notably IB05 (3 F), who had truncus arteriosus repair with a bovine conduit and exhibited the strongest Neu5Gc response despite early age. Similarly, IB06 (15 F) and IB07 (14 M), both with recent revisions involving bovine or porcine grafts, displayed elevated titers. In contrast, IB01 (10 M), IB02 (52 M), and IB03 (9 M) had low or undetectable anti‑Neu5Gc titers, suggesting either reduced immunogenic priming, tolerance, or long elapsed time since graft implantation, allowing declining antibody levels.

In contrast, anti‑αGal titers, which target the well‑defined Galα1-3Gal epitope present in porcine-derived tissues [30-32], were highest in the adult patient IB02 (52 M), who had undergone a Ross procedure with porcine graft over a decade earlier, indicative of chronic exposure and durable immune memory. Pediatric and adolescent patients (IB03-IB07) exhibited moderate and consistent αGal titers regardless of sex, underscoring the ubiquitous immunogenicity of αGal across xenograft types. IB01 remained the lowest in both antibody categories, suggesting minimal immunological activation or a potential regulatory advantage.

These observations reflect the existing literature: Neu5Gc is highly immunogenic in xenograft contexts, contributing significantly to allo‐inflammatory responses [33] while αGal, a single epitope antigen, elicits broadly consistent immune responses in humans [31]. The variable and high anti‑Neu5Gc titers in younger patients, particularly females, raise the possibility of sex-based immune differences and draw attention to the antigenic complexity of Neu5Gc linked to multiple glycan backbones [32]. In contrast, αGal responses appear relatively uniform, likely driven by early life microbiota exposure and inherent natural antibody production.

Notably, patients requiring redo surgeries in adolescence IB04 and IB06 also showed higher anti‑Neu5Gc titers, intimating a possible association between immune sensitization and conduit degeneration or dysfunction. This parallels findings that anti‑Neu5Gc antibodies can mediate endothelial activation, complement deposition, and chronic inflammation, collectively termed “xenosialitis,” which may compromise graft integrity [31-33]. Similarly, high-affinity anti‑αGal antibodies are known to induce complement-dependent cytotoxicity and antibody‑dependent cell-mediated cytotoxicity (ADCC), triggering endothelial injury and xenograft rejection [33,34]. Alternate strategies for xenograft decellularization include the use of sodium dodecyl sulfate (SDS), a combination of SDS with sodium deoxycholate (SD), Triton X-100, and repeated freeze-thaw cycles. Among these, protocols incorporating freeze-thaw cycles for the decellularization of bovine pericardium have demonstrated greater efficiency in removing residual DNA, eliminating the α-Gal antigen, and minimizing cytotoxic effects [35].

Clinically, this cohort demonstrates that younger patients with bovine conduit exposure may mount robust anti‑Neu5Gc responses, even in early childhood, contradicting assumptions of pediatric immune immaturity. Conversely, adult patients may accumulate higher αGal titers over time. These differential trajectories highlight the need for individualized immuno-monitoring post-xenograft implantation. Integration of antibody profiling into routine follow-up could assist in identifying patients at risk of graft degeneration or immune-mediated complications, informing surveillance intervals and biomaterial choice in future surgeries.

Collectively, our data reinforce the significance of both Neu5Gc and αGal immunogenicity in xenografts and suggest that sex, age at implantation, graft material, and time since surgery modulate the immune response. Monitoring both antibody classes may serve as valuable biomarkers for assessing long-term graft viability and optimizing patient‑specific follow‑up strategies.

Limitations

Selection Bias in Benchmark

Setting a four- to five-year period as the benchmark may exclude relevant early or late graft failures, potentially skewing durability assessment toward mid-term outcomes only. Patients lost to follow-up before the four-year point might have unreported complications.

Limited Generalizability

The four-year threshold may not apply to all patient populations (eg, pediatric vs. adult) due to differences in growth rates, hemodynamic stress, and immune responses.

Potential Confounding Variables

Variations in surgical techniques, post-operative management, and patient comorbidities could influence graft performance independently of time since implantation.

Small Sample Size

In this study, only seven serological samples were compared with a single reference sample from a healthy donor. Furthermore, only one explanted graft was available for investigation.

Incomplete Immunological Data

Serological markers like anti-alpha Gal or anti-Neu5GC antibodies were not consistently measured at six-month intervals after xenograft implantation, limiting insight into immune-mediated degeneration.

## Conclusions

Our study demonstrates that indigenously developed decellularized xenografts provide favorable postoperative outcomes. Their acellular, non-thrombogenic, biocompatible, and non-cytotoxic characteristics facilitate seamless integration with native tissues while reducing the risks of immunogenic rejection, fibrosis, and calcification. These grafts also exhibit adequate mechanical strength to withstand high blood pressure, supporting their potential application in vascular reconstruction surgeries and ventricular aneurysm repair. In addition, our simplified and cost-effective decellularization protocol for bovine pericardium generates an extracellular matrix microenvironment conducive to host cell recellularization, re-endothelialization, and neovascularization. Although the study is limited by a small sample size, the findings provide important insights into the incidence and levels of α-Gal and Neu5Gc antibodies in recipients of decellularized xenografts.
