# Correction: Serological Assessment of Alpha Galactosidase, N-Glycolylneuraminic Acid, and Histopathological Observations in Xenograft Recipients

**DOI:** 10.7759/cureus.c301

**Published:** 2025-09-19

**Authors:** Balasundari Ramesh, Sowmya Ramanan, Balaji Srimurugan, Adegbenro O Fakoya

**Affiliations:** 1 Cellular Biology and Anatomy, Louisiana State University Health Sciences Center, Shreveport, USA; 2 Cardiothoracic Surgery, Sree Chitra Tirunal Institute for Medical Sciences and Technology, Trivandrum, IND; 3 Cardiovascular Surgery, Amrita Institute of Medical Sciences and Research Centre, Chennai, IND

In the abstract, the immunogenic epitope was incorrectly referred to as “alpha-galactosidase (α-gal)”. The correct terminology is “galactose-α-1,3-galactose (α-Gal)”, which is the carbohydrate structure recognized by anti-Gal antibodies and relevant to xenotransplantation. Alpha-galactosidase is an enzyme and not the epitope itself.

